# Respiratory Physiotherapy Intervention Strategies in the Sequelae of Breast Cancer Treatment: A Systematic Review

**DOI:** 10.3390/ijerph19073800

**Published:** 2022-03-23

**Authors:** Maria Jesus Vinolo-Gil, Rocío Martín-Valero, Francisco Javier Martín-Vega, Manuel Rodríguez-Huguet, Veronica Perez-Cabezas, Gloria Gonzalez-Medina

**Affiliations:** 1 Department of Nursing and Physiotherapy, University of Cadiz, 11009 Cadiz, Spain; mariajesus.vinolo@uca.es (M.J.V.-G.); javier.martin@uca.es (F.J.M.-V.); manuel.rodriguez@uca.es (M.R.-H.); veronica.perezcabezas@uca.es (V.P.-C.); gloriagonzalez.medina@uca.es (G.G.-M.); 2 Rehabilitation Clinical Management Unit, Interlevels-Intercenters Hospital Puerta del Mar, Hospital Puerto Real, Cadiz Bay-La Janda Health District, 11006 Cadiz, Spain; 3 Institute for Biomedical Research and Innovation of Cádiz, 11009 Cadiz, Spain; 4 Department of Physiotherapy, Faculty of Health Science, Ampliacion de Campus de Teatinos, University of Malaga, C/Arquitecto Francisco Peñalosa 3, 29071 Malaga, Spain; 5 Biomedical Research and Innovation Institute of Cadiz (INiBICA), Research Unit, Puerta del Mar University Hospital, University of Cadiz, 11009 Cadiz, Spain; 6 CTS-986 Physical Therapy and Health (FISA), University Institute of Research in Social Sustainable Development (INDESS), 11009 Cadiz, Spain

**Keywords:** breast neoplasms, cancer treatment protocols, physiotherapy, breathing exercises, complications

## Abstract

Breast cancer treatments can trigger respiratory sequelae. Respiratory physiotherapy helps to eliminate or mitigate the sequelae by optimizing respiratory function. This systematic review aims to synthesize the scientific evidence and assess its quality regarding the use of respiratory physiotherapy in the sequelae of breast cancer. The Cochrane Library, Physiotherapy Evidence Database, PubMed, Web of Science, Scientific Electronic Library Online, Cumulative Index of Nursing and Allied Literature Complete, and Scopus were searched. Study quality was determined using the PEDro scale, STROBE Statement, and Single-Case Experimental Design Scale. Ten studies, six clinical trials, one case study, and three observational studies were selected. The mean methodological quality of the clinical trials was 5.6, that of the case study was 7, and that of the observational studies was 56%. Respiratory physiotherapy has been observed to improve respiratory capacity, lung function, respiratory muscle strength, effort tolerance, dyspnea, fatigue, thoracic mobility, upper limb volume, sleep quality and quality of life, as well as sensitivity to adverse physiological reactions, nausea, vomiting, and anxiety. However, it is not effective for vasomotor symptoms. More clinical trials are needed. These studies should homogenize the techniques used, as well as improve their methodological quality.

## 1. Introduction

Breast cancer (BC) is the most common malignant tumors in women worldwide, with the exception of skin cancer [1].

According to the World Health Organization, more than one million new cases are diagnosed each year, accounting for almost a quarter of all malignant tumors in women and affecting one in 100 men. In the West, it has been shown that one in nine to twelve women will suffer from the disease in her lifetime [2].

The incidence rate is increasing. This is indicative of earlier detection, as the mortality rate has not increased at the same rate. This seems to be due to the fact that, together with early diagnosis, treatment intervention is more appropriate to the pathology and the patient, although it is one of the main causes of death from cancer among women in developed countries [3].

However, with improved survival rates, more patients are facing persistent treatment-related symptoms. These treatments can be surgical, systemic (hormonal therapy and chemotherapy), and radiotherapy which, in turn, can have adverse effects on the respiratory system [4].

With regard to the consequences of surgical treatment, it can be found related to immobilization and postoperative injury, including respiratory disorders, reduced mobility of the thorax due to postoperative pain, circulatory disorders, as well as reduced muscle strength and alteration of the cough reflex [5]. In addition, in the case of radical mastectomy, it can lead to disturbances in body posture, causing winged scapulae, ascended shoulders, and increased curvature of the cervical and thoracic spine [6], reducing thoracic and fascial mobility, disturbing ventilatory mechanics, and impairing the functions of the respiratory system [7]. This leads to a reduction in the mobility of the thorax, resulting in reduced respiratory muscle efficiency and fatigue, as well as a decrease in the range of motion of the diaphragm [5].

There is evidence that chest radiation may affect the cardiorespiratory capacity of women with breast cancer due to reduced maximal oxygen consumption compared to healthy people [7].

In addition, incidental exposure to the heart may occur, increasing the risk of coronary heart disease and cardiovascular mortality [8]. Thoracic radiotherapy also decreases respiratory and exercise capacity, probably due to restricted chest wall mobility [9]. There may also be risks of lung parenchymal damage [10,11], loss of type II pneumocytes, and loss of surfactant and basement membrane edema impacting respiratory function and impairing the ability to perform physical activities [12]. It can progress to pulmonary fibrosis that induces a restrictive pattern.

Finally, hormone therapy with tamoxifen and some chemotherapy drugs can also contribute to the appearance of pulmonary toxicity [13,14], decreasing pulmonary function tests such as forced vital capacity, forced expiratory volume in 1 s, total lung capacity, peak expiratory flow at 50% and 25% of vital capacity, and carbon monoxide diffusion capacity [15]. In addition, there is peripheral muscle weakness [16] and respiratory muscle weakness with increased exercise intolerance [17,18].

These findings highlight the importance of respiratory muscle function, especially with regard to exertional dyspnea and reduced exercise performance reported in breast cancer [19].

Physiotherapy is an integral part of treatment for breast cancer patients. It allows patients to regain physical fitness and reduce the side effects of treatment. Respiratory physiotherapy (RP), which consists of a combination of strategies aimed at preventing, treating, and stabilizing cardiorespiratory disorders in adult and pediatric patients [20], is an accepted method to maintain and improve respiratory capacity, quality of life, and post-treatment sequelae of breast cancer [21].

RP has been shown to be helpful in other types of cancer, such as lung cancer [22].

There are numerous studies related to physiotherapy for lymphoedema [23,24,25,26,27], pain [28,29,30,31,32], restoration of shoulder mobility [33,34], and physical training [35,36,37,38]. However, they do not take into account respiratory and other harmful symptoms associated with treatments used in breast cancer.

Therefore, the aim of our study is to synthesize the scientific evidence and assess its quality regarding the use of intervention strategies in RP in the aftermath of breast cancer. It also aims to know the use of respiratory physiotherapy on the negative effects of the treatments used in breast cancer, as well as to know the techniques used.

## 2. Materials and Methods

A systematic review and meta-analysis was conducted and recorded in PROSPERO (CRD42021227590) using the Preferred Reporting Items for Systematic Reviews and Meta-analysis (PRISMA) [39]. The PRISMA checklist is detailed in Appendix A.

### 2.1. Search Strategy

The search was conducted from September to October 2020 in the following databases: The Cochrane Library, Physiotherapy Evidence Database (PEDro), PubMed, Web of Science, Scientific Electronic Library Online (SciELO), Cumulative Index of Nursing and Allied Literature Complete (CINAHL), and Scopus.

“Breast Neoplasms”, “physiotherapy”, “breathing exercises”, “breast cancer”, “physical therapy”, “rehabilitation”, “respiratory muscle training”, were used as keywords. These were combined with AND and OR.

### 2.2. Eligibility Criteria

Eligibility criteria were based on the PICO framework [40]: (P) Participants, over 18 years of age diagnosed with breast cancer who had received adjuvant therapy, after surgery; (I) Intervention, any patient that had undergone any technique within the field of respiratory physiotherapy defined as combination of strategies aimed at preventing, treating, and stabilizing cardiorespiratory disorders in adult and pediatric patients [21]; (C) Comparison, no treatment, placebo, or other intervention; Outcome, any clinical variable that could be improved following respiratory physiotherapy treatment.

No limitations were made in terms of language. Regarding the design of the study, all types of study designs were considered. The search was limited to the last 10 years.

Studies where the intervention was aerobic or resistance exercise programs, breathing exercises in yoga, qigong, tai chi, and pilates, and those where the patient was receiving palliative treatment, were excluded.

### 2.3. Study Selection Process and Data Extraction

The papers were independently reviewed and selected by two of the researchers. The final result was agreed with a third investigator.

The information extracted from each study was related to authors, number and characteristics of the sample, specific treatment used for cancer, type of respiratory physiotherapy, duration of treatment, outcome measures, measurement instrument, and results obtained.

### 2.4. Assessment of Methodological Quality

To assess the methodological quality of the clinical trials, the PEDro scale that is based on the Delphi list developed by Verhagen et al. [41] was used: (item 1) specified choice criteria, (item 2) random allocation, (item 3) covert allocation, (item 4) baseline similarity, (item 5) subject blinding, (item 6) therapist blinding, (item 7) assessor blinding, (item 8) more than 85% follow-up for at least one key outcome, (item 9) intention-to-treat analysis, (item 10) statistical comparison between groups for at least one key outcome, and (item 11) point measures and variability for at least one key outcome. Item 1 is not scored. It is scored 1 when the condition is met and 0 when it is not met.

The PEDro scale categorizes clinical trials as “good” quality (score 6–10), “fair” quality (score 4–5), and “poor” quality (score < 4) [42].

For observational studies, the STROBE Statement was used: it looks at the quality of information from observational studies with a focus on prevalence (cut-off, case-control, cross-sectional). It consists of 22 items on the title of articles, abstract, introduction, methods, results, discussion sections, and other information. A total of 18 items are common to all three designs; the other items are design-specific. For some items, information should be given separately for cases and controls in case-control studies, or exposed and non-exposed groups in the cross-sectional study and cross-sectional studies [43].

For the assessment of case studies we used the Single-Case Experimental Design Scale (SCED) which includes 11 items, of which 10 are used to assess methodological quality and the use of statistical analysis [44]. An additional item (specification of the clinical history) is included which is not scored. The items are (item 1) clinical history, (item 2), target behaviors, (item 3), design, (item 4) baseline, (item 5) sampling behavior during treatment, (item 6) raw data record, (item 7) inter-rater reliability, (item 8) independence of assessors, (item 9) statistical analysis, (item 10) replication, and (item 11) generalization.

A dichotomous response format (present/absent) is used, with 1 point if the criterion has been met. Thus, the score ranges from 0 to 10, with higher scores indicating better methodological quality.

### 2.5. Risk of Bias of Included Studies

The risk of bias was calculated for each study selected using the Cochrane Collaboration Tool [45]. The following types of bias were assessed: selection bias, performance bias, detection bias, attrition bias, reporting bias, and other bias. Two reviewers (M.J.V.-G. and R.M.-V.) assessed the methodological quality and the risk of bias of the studies. In case of doubt, authors resolved disagreements by consensus and consulting a third author (G.G.-M.) when necessary.

## 3. Results

This section may be divided by subheadings. It should provide a concise and precise description of the experimental results and their interpretation, as well as the experimental conclusions that can be drawn.

### 3.1. Selection of Studies

**Figure 1:**
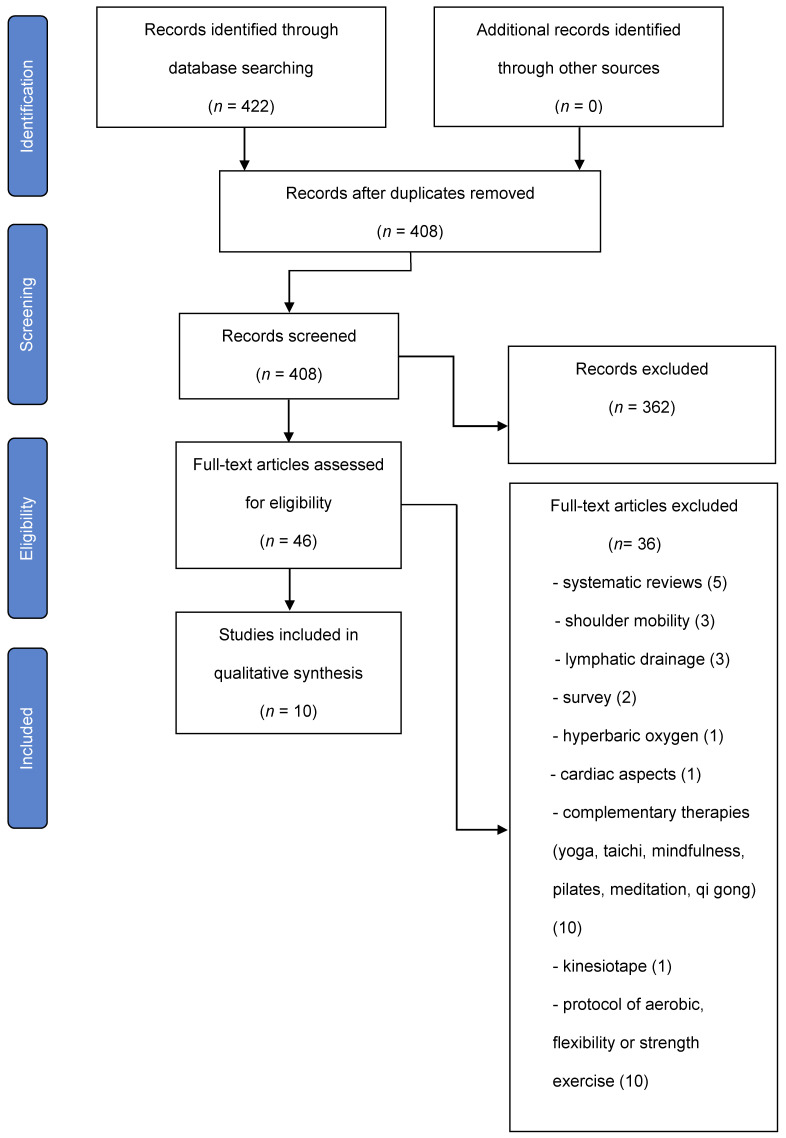
Flow diagram.

The entire selection process in the different phases is detailed in a PRISMA flow chart (Figure 1).

The main characteristics of the studies are shown in Table 1.

### 3.2. Data Extraction

The sample consisted of 908 patients, where only 8% were male [50,51]. The number of subjects participating in the studies ranged from 5 [49] to 315 [51] persons and the age of the participants ranged from 54 [46] to 62.5 [55] years, with a mean age of 54. Regarding the type of surgery, radical mastectomy [47,49,51,52,54,55], segmentectomy [49,51,55], and axillary lymphadenectomy [49,51,55] were performed.

In reference to the stage of the cancer, according to the tumor-node-metastasis (TNM) staging system: “stage” I [48,53] and II [47,48,49,52,53] were used. Only one article specified tumor size [52]: 2.5 ± 1.6 (0.1–9) cm^3^. In the studies in which radiotherapy was used and its doses were specified, it was found that the most commonly used dose was between 40 Gy [47] and 50 Gy [49,52].

All patients underwent surgery, 12% also received radiotherapy and chemotherapy, 42% radiotherapy, 17% chemotherapy, and less than 1% received radiotherapy, chemotherapy, and hormone therapy.

In three of the studies, the sample also consisted of lung cancer patients [50], menopausal women [53], or people with cancer in the abdominal region [51].

Regarding the RP intervention strategies used, there is a lot of variability. RP was used as a sole treatment [50] or was combined with other exercises [47,51,54,55].

Among the RP interventions used were the use of the incentive spirometer and PEP mask [49], respiratory muscle training [56], and the lumbar quadratus lumborum muscle energy technique [47]. In one of the studies the technique or techniques used were not specified [52].

In four of them [47,51,54,55], RP was performed within a broader protocol in combination with other techniques, such as muscle relaxation [51,54], guided imagery and music [51], soft tissue therapy on muscle fascia and postoperative scar [47], and upper limb exercises [47].

Interventions used in the control groups included aerobic exercise [48], nursing care [46,54], or “no training” or rapid shallow breathing exercises [53].

Concerning the number of sessions, frequency and total duration of treatment was very heterogeneous, ranging from 5 times a week, 5 times a day, 30 min [53] to a single session of 45–60 min [51].

The variables studied were inconsistent except for spirometric data [47,48,49,50,52]. Parameters related to respiratory alterations such as fatigue [49,50], effort tolerance [49,50], aerobic capacity [50], dyspnea [50], quality of life [50], and thoracic mobility [47] were evaluated. Vasomotor symptoms [53], nausea [46], vomiting [46], satisfaction [51], pain perception [47], anxiety [54], upper limb volumen [55], mood [53], sleep disturbances [53], and functional status [46] were also assessed.

Regarding the measurement instruments, there is little homogeneity. Spirometric data [47,48,49,50,52] such as FEV1, FVC, and VVM were used to assess lung function; PIM and PEM to assess respiratory muscle strength [48]; Borg scale [49] and FACIT-F [49,50] to measure fatigue; 6 MWT to assess exercise tolerance [49,50]; MRC, BDI, and TDI to measure dyspnea [50]; TUG to assess lower limb mobility and risk of falls [50]; SF-36 [50], QOL37 [50], and MCGill quality of life questionnaire [55], RSCL [54] to measure quality of life; VAS to measure pain [47], STAI to assess anxiety [54], and PANAS and HFRDIS to assess hot flushes [53].

Variables related to upper limb symptomatology [55] were assessed by perimetry, bioimpedance and tonometry, mood [53] with POMS-SF, sleep quality [53] with PSQI and functional status with FLI-C [46].

In terms of outcomes, there were improvements in lung function [47,48,49,50,52], respiratory muscle strength [48,50], exercise tolerance [49], dyspnea [50], fatigue [49], thoracic mobility [47], upper limb volumen [55], sleep quality [53], and quality of life [50,51], as well as a reduction in sensitivity to adverse physiological reactions [54], number of vomiting [46], nausea [46], and anxiety [54].

### 3.3. Methodological Quality Assessment

The results of the quality assessment of the different studies are shown in Table 2, Table 3 and Table 4. Table 2 presents the methodological quality of the clinical trials. Table 3 and Table 4 show the methodological quality of the observational studies and the case study, respectively.

The mean methodological quality of the clinical trials as measured by the PEDro scale was 4.5, that of the case study as measured by the SCED scale was 7, and in the case of the observational studies, 56% of the recommendations of the STROBE Statement were met.

### 3.4. Risk of Bias of Included Studies

**Figure 2:**
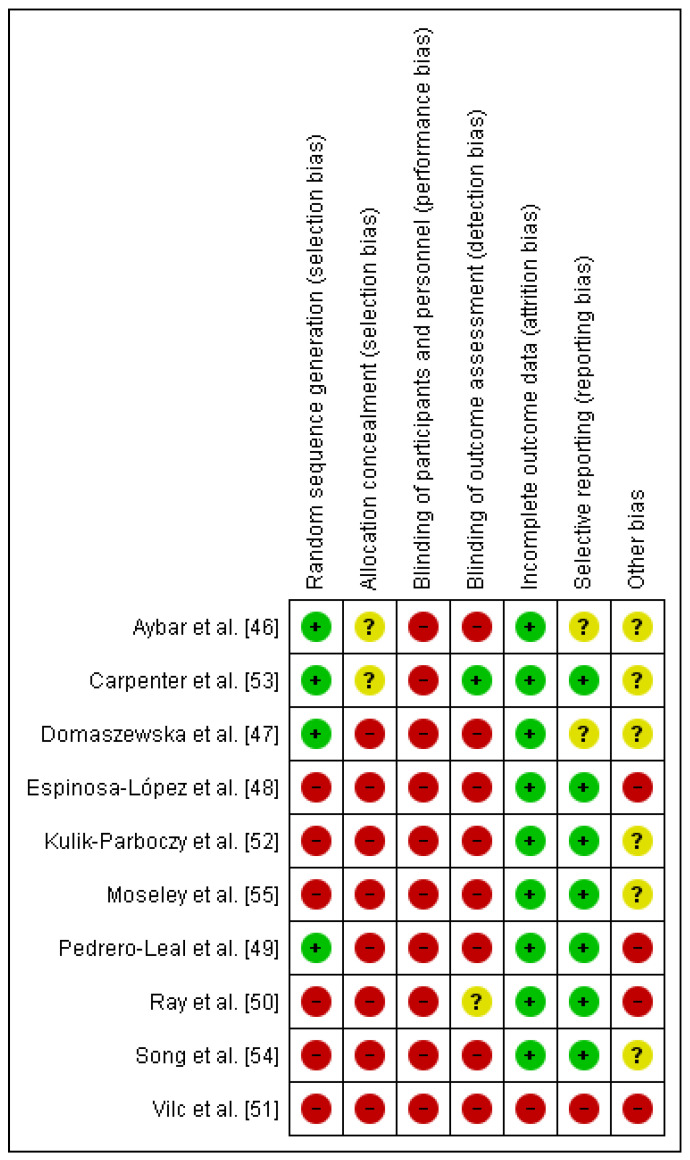
Risk of bias summary [46,47,48,49,50,51,52,53,54,55].

**Figure 3:**
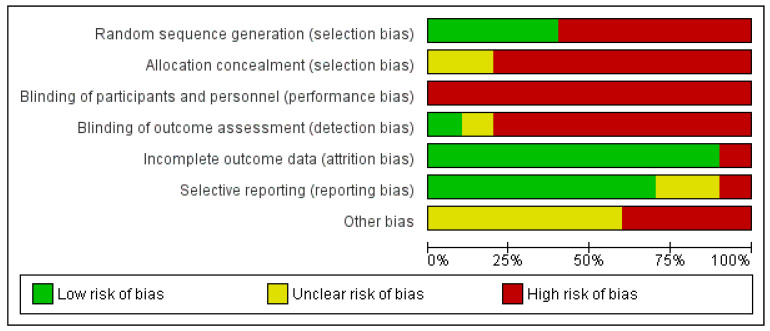
Risk of bias graph.

The Cochrane Risk of Bias Assessment Tool was used to assess the risk of bias of the articles included in this review. The results of the risk of bias can be observed in Figure 2. It should be noted that the risk of bias is high in relation to performance bias and detection bias because patients and therapists were not blinded, and in only one article were the evaluators blinded [53]. The risk of bias is also high in relation to selection bias because there was random sequence generation in only four of the trials [46,47,49,53]. With respect to attrition bias, all of the them were low-risk, except one [51] (Figure 3).

## 4. Discussion

A systematic review was carried out to synthesize the scientific evidence and evaluate its quality regarding the use of RP intervention strategies in the treatment of the sequelae of breast cancer.

### 4.1. Characteristics of the Sample

With regard to the characteristics of the sample, it was homogeneous in terms of sex, as the number of women was always higher than men. This is due to the fact that breast cancer affects women to a greater extent [56,57]. According to the age of the participants, the sample was heterogeneous, as was the specific treatments previously received by the study participants. Of the 10 studies, all had surgery, 5 had radiotherapy [47,49,51,52,53], 5 had chemotherapy [46,49,52,53,54], and 2 had hormone therapy [47,49]. Adjuvant radiotherapy is the standard treatment following breast conserving surgery in early breast cancer [58].

### 4.2. Measuring Instruments

Concerning measuring instruments, the most commonly used in our paper was spirometry, which is the main pulmonary function test, fundamental for the evaluation and follow-up of respiratory diseases [59], coinciding with other studies in which pulmonary function in breast cancer was also measured with this test [60,61].

### 4.3. Intervention Strategies in RP

The most commonly used techniques in RP in general are drainage of secretions, mobilization of the rib cage, and ventilatory techniques [21].

In comparison with our review, we would agree with the ventilatory techniques [49], since, given the type of alteration that occurs in breast cancer, secretion drainage would not be the technique of choice in principle.

Within the ventilatory techniques we can find the thoracic mobility that may increase the vital capacity in patients with chronic respiratory disease [62].

In one of the trials in our review [47], the aim was to improve thoracic mobility, but this was not achieved through specific mobilization of the thorax, but rather with respiratory and circulatory exercises and soft tissue therapy, the latter being used in several of the studies evaluated in this document to treat muscle fascia and postoperative scarring [47,48]. However, among the techniques, the most widely used was deep diaphragmatic breathing exercises [47,51,53,54,55].

In some of the papers in this review, breathing exercises have been combined with other techniques, such as muscle relaxation [51,54], coinciding with other trials carried out in other cancer populations, in which anxiety and emotional distress were reduced [63,64]. Along the same lines, Stoerkel et al., using guided mind-body techniques (breathing, meditation, guided imagery, self-hypnosis suggestions), obtained improvements in pain, nausea, sleep, fatigue, global health, and quality of life after surgery in breast cancer [65].

These latter aspects can also be improved in people with cancer, who are undergoing treatment, through physical training [66], which we know is a mainstay of RP, but is not the focus of this manuscript.

Another study used breathing exercises together with soft tissue techniques, which has also been used as the sole technique, in post-mastectomy patients to eliminate muscle and fascia stiffness in the postoperative scar area [67]. Other therapeutic methods, which are not commonly used, but could be applicable to improve the functions of the respiratory system by restoring the correct mobility of the thorax and improving the work of the respiratory muscles in the operated area, are thoracic rib and joint mobilization, trigger point therapy, and kinesiotaping [68].

Breathing exercises are widely used in breast cancer within broader interventions such as yoga or telerehabilitation platforms.

Considering the effects of therapeutic yoga in breast cancer, yogic breathing (pranayama) has shown numerous beneficial health effects in breast cancer patients undergoing radiotherapy [69] or chemotherapy [70], with an improvement in quality of life and fatigue [71], findings also found in our review. These authors suggested, as a possible cause, that during controlled breathing exercises, the stretching of lung tissue produces inhibitory signals in the vagus nerve, which ultimately shifts the autonomic nervous system towards the parasympathetic domain, resulting in a calm and alert state of mind, coinciding with our review where a reduction in sensitivity to adverse physiological reactions and anxiety [54] was found.

In terms of telerehabilitation, the e-CUIDATE platform provides access to a range of content such as breathing, mobility, strength, and stretching exercises to breast cancer patients during adjuvant treatment, achieving improvements in terms of functional and cognitive performance in breast cancer survivors, as well as decreasing cost and increasing accessibility [72].

### 4.4. Main Results

The usefulness of RP has been observed in the improvement of pulmonary function [47,48,49,50,52], respiratory muscle strength [48,50], effort tolerance [49], dyspnea [50], fatigue [49], thoracic mobility [47], upper limb volumen [55], sleep quality [53], and quality of life [46,50,51], as well as a reduction in sensitivity to adverse physiological reactions [54], nausea [46], vomiting [46], and anxiety [54]. However, it is not useful in improving vasomotor symptoms [53].

Where it seems to be most effective is in respiratory capacity, as improvements were found in spirometric data such as FEV1, CV, CVF, MVVV, and in the improvement of muscle strength of the respiratory muscles, improving fatigue, dyspnea, and mobility of the rib cage. These results coincide with those obtained in other types of cancer, such as lung cancer, achieving a significant decrease in the severity of dyspnea and fatigue, although they were not significant in respiratory capacity [73], contrasting the latter finding with that obtained in our review.

This variable has been studied through spirometric data from spirometry and in only one of the articles was the Peak Exercise Test with a cycle ergometer applied to the study participants to determine the VO_2_ peak [50]. In other populations, such as heart patients or athletes, its use is very frequent [74].

Breast cancer patients suffer from impaired respiratory capacity as measured by VO_2_ peak [75]. Poor VO_2_ peak is associated with poorer quality of life [76] and increased morbidity and mortality in cancer survivors [77], and may be an independent predictor of survival in metastatic disease [78].

In relation to the above, there is also a decrease in inspiratory and inspiratory muscle function in these patients [19]. In only one study in our review, training of PIM and PEM was performed [48], but their training would be crucial to improve O_2_ consumption [56].

For all these reasons, it is striking that there is very little research related to respiratory physiotherapy that takes these reflections into account and we recommend that future studies include the measurement of this variable, as well as specific training of the respiratory musculature.

It is worth mentioning the only study in our review that found some important correlations between the results obtained and the type of intervention [52]. Kulik-Parobczy et al. found differences in means of spirometric indicators before and after rehabilitation, especially in patients who underwent mastectomy and lymph node status, radically reducing the level of PEF by as much as 64 units. Other findings included were a positive influence of chemotherapy on the spirometric indicator before and after rehabilitation and a significant impact of the rehabilitation on FEV_1_

Another variable studied by 30% of the studies in our review was quality of life [46,50,51], obtaining positive results, coinciding with the Cochrane’s review that evaluated the quality of life in breast cancer patients after physical activity. Their trials had as low methodological quality as those reviewed in this manuscript [66].

On the other hand, it is a treatment that does not require large investments in technology [79] and it could easily be implemented in patients with sequelae of breast cancer treatment. Even so, its use is not widespread [21] and it is being underutilized, although it is in demand by the patients themselves [80].

### 4.5. Strengths and Limitations

The present study has several strengths, including the broad and easily reproducible search strategy applied to seven major medical databases. In addition, studies have been systematically selected by applying well-defined inclusion/exclusion criteria. However, there are several limitations that need to be addressed before drawing conclusions from the results of the present analysis. Heterogeneity among the different studies was so extensive that a meta-analysis could not be performed.

There was little uniformity in study populations (some of them were not unique to breast cancer patients), sample sizes, RP interventions and their duration, measured variables, and different measurement instruments.

Despite a thorough search, the literature found was sparse: there were only five clinical trials with “fair” scores, one case study, and three observational studies that met half of the recommendations of the STROBE Statement. One of the reasons for the low scores of these studies could have been the low baseline similarity and the use of single blinding, due to the inherent nature of the studies in clinical trials, and in the case of the observational and case studies, the low external validity which would make it difficult to generalize the results. For all the above reasons, positive results should therefore be interpreted with caution.

More studies are needed to prove the efficacy of RP so that it can be more widely used in breast cancer, given all the problems associated with its treatment, as well as more in-depth research to broaden the therapeutic options for this type of patient, including determining which type of treatment (radiotherapy, chemotherapy, surgery, hormone therapy) could be more effective. In addition, it could be convenient to investigate whether respiratory physiotherapy can help not only the complications arising from the treatment but also the treatment itself.

## 5. Conclusions

In conclusion, it is observed that respiratory physiotherapy is not widely used in the sequelae of breast cancer treatment. Respiratory physiotherapy improves lung function, exercise tolerance, dyspnea, fatigue, thoracic mobility, upper limb volume, sleep quality, functional status, and quality of life, as well as reducing sensitivity to adverse physiological reactions, nausea, vomiting, and anxiety. RP is not effective in improving vasomotor symptoms. In terms of RF interventions, diaphragmatic deep breathing exercises were the most commonly used.

This review confirms the limited evidence in favor of the benefits of these RP intervention strategies for breast cancer sequelae.

Future studies with low risk of bias are required to determine the respiratory physiotherapy techniques needed to improve specific outcomes among women who have undergone surgical treatment and adjuvant therapy.

## Figures and Tables

**Table 1:** Characteristics of the study intervention.

Author, (Year)	Intervention	Outcomes	Measuring Instruments	Results
Aybar et al. (2020) [46]	CG ^1^: nursing care IG ^2^: breathing exercise	- Nausea severity - number of nausea, vomiting, retching episodes - Functional status - Hours of breathing exercises	- VAS ^3^ - patient diary - FLI-C ^4^	- Lower number of nausea, vomiting, and retching episodes and experienced lower severity of nausea (*p* < 0.05) in IG. - Nausea: IG 0.17 (0.30) vs. CG 0.15 (5.09); Z = −5434; *p* = 0.001) - Vomiting: IG 0.00 (0.0) vs. 0.36 (1.2); Z = 3003; *p* = 0.003) - Retching: IG 0.0583 (0.19) vs. CG 0.85 (1.7); Z = −2613; *p* = (0.009) - FLI-C score: IG vs. CG: 11.05 ± 16.35/−11.86 ± 15.15; Z: −4716; *p* = 0.001)
Domaszewska et al. (2019) [47]	CG/IG: 1st month after surgery: gradual verticalization, circulatory exercises, breathing exercises 3 times/day (5–6 repetitions) supervised by physiotherapist. self-assisted exercises of 10–15 min 5–10 v CG: 2nd–12th month: self-assisted conditioning exercises and breathing exercises (30 min 2 times/day). GI: 2nd–12th month: the same + soft tissue therapy on muscle fascia and postoperative scar (2 times/week)	- Respiratory capacity - Thorax mobility - Pain	- spirometry - Assessment of the mobility of thorax with tape measure - VAS	Improved respiratory capacity - VC ^5^: IG: 11.53 ± 18.03 (CI: 102.64–117.86–6.29) vs. CG: 86.72 ± 17.46 (CI: 79.51–93.03); (*p* < 0.001); CI: confidence intervals 95% - FEV_1_^6^: IG: 111.92 ± 17.17 (CI: 104.67–119.17) vs. CG: 88.60 ± 17.09 (CI: 81.55–95.65); (*p* < 0.001); CI: confidence intervals 95%. - FEV_1_/FVC ^7^: IG: 104.79 ± 8.93 (CI: 101.02–108.56) vs. CG: 112.40 ± 8.16 (CI: 109.03–115.77); (*p* < 0.01); CI: confidence intervals 95%. - MVV ^8^: IG: 101.54 ± 21.57 (CI: 92.43–110.65) vs. CG: 72.64 ± 20.75 (CI: 64.07–81.21); (*p* < 0.001); CI: confidence intervals 95%. Improved thorax mobility IG: 5.90 ± 0.92 (CI: 5.51–6.29) vs. CG: 3.00 ± 1.09 (CI: 2.55–3.45); (*p* < 0.001); CI: confidence intervals 95%.
Espinosa-López et al. (2019) [48]	CG: aerobic exercise IG: same + TEM ^9^ quadratus lumbar muscle	- Respiratory muscular strength	- Spirometry - PIM ^10^, PEM ^11^	Improved MIP, MEP; the mean change in MIP was 68% and in MEP, 57%; (*p* < 0.05)
Pedrero-Leal et al. (2019) [49]	- incentive spirometer - PEP ^12^ mask	- Respiratory capacity - Effort tolerance - Perceived fatigue	- Spirometry - Borg scale - 6MWT ^13^ - FACIT-F ^14^	Improved CV, FEV_1_, fatigue and distance in 6MWT
Ray et al. (2017) [50]	- TMR ^15^ - 3 sets (15 repetitions) with a resistance of 40% to 70% in the 4th week	- Respiratory capacity - Respiratory muscle strength - Dyspnea - Quality of life - Fatigue - Cycloergometer stress test	- Spirometry - MRC ^16^, BDI ^17^; TDI ^18^ - TUG ^19^ - 6MWT - SF36 ^20^, QOL37 ^21^ - FACIT-F ^22^	- SF36: significant improvement in physical health scale (*p* = 0.039) MIP and MEP increased 29% ± 21% and 34% ± 32%, respectively (*p* < 0.001). Submaximal endurance time (16.9 ± 7.4 min vs. 31.4 ± 7.7 min, *p* = 0.001), the distance covered in the 6MWT (427 ± 84 m vs. 471 ± 95 m, *p* = 0.005), dyspnea index (6.4 ± 1.0 vs. 7.6 ± 1.3, *p* = 0.02), and QOL (total 85.3 ± 9.4 vs. 97.8 ± 12.7, *p* = 0.014)
Vilc et al. (2019) [51]	- Diaphragmatic Deep Breathing Exercises - PMR 20 min of 10 muscle groups - Guided imagery and music Groups of 4–5 people	Satisfaction with the program	Likert-type survey	Improvement of quality of life by subjective impression of the patient studied by Likert-type questionnaire.
Kulik-Parobczy et al. (2019) [52]	Respiratory physiotherapy (technique not specified)	- Respiratory capacity	Spirometry	Improved lung age and FEV_1_% by 1.8 units per day of treatment (*p* < 0.0001). Particularly evident in patients with more advanced cancer stages.
Carpenter et al. (2013) [53]	- IG: slow, deep diaphragmatic breathing training 6–8 breaths per min, 2 times/day, 15 min. Breathing at onset of flushing - Control IG: fast shallow breathing training - CG: no training	- Frequency, severity and vasomotor symptoms of hot flushes - Interference of hot flushes with daily life - Management of hot flushes - Mood - Sleep disturbances	- HFRDIS ^22^ - PCI ^23^ - PANAS ^24^ - POMS-SF ^25^ - PSQI ^26^	- Significant difference in global PSQI (*p* = 0.20) - Slow deep diaphragmatic breathing was not significantly more effective than control or usual care breathing on vasomotor symptoms (*p >* 0.05).
Song et al. (2013) [54]	- CG: nursing care - IG: same + muscle relaxation training and controlled abdominal breathing exercises (6 times per minute or 15 s per breath)	- Anxiety - Psychological and physiological discomfort - Quality of life	- STAI ^27^ - RSCL ^28^	- Reduced sensitivity to adverse physiological reactions (decreased appetite, decreased energy, nausea, cough, mouth ulcers, gastric reflux, decreased back pain) - Decreased anxiety: IG: 39.1 ± 4.5 vs. CG: 46.2 ± 6.0; F value 21.202; *p* = 0.001 - Decrease Physiological dimension: IG: 42.8 ± 4.6 vs. CG: 54.5 ± 5.8; F value 71.116; *p* = 0.001 - Decrease Psychological dimension: IG: 15.2 ± 2.2 vs. CG: 18.7 ± 3.1; F value: 24.291; *p* = 0.001
Moseley et al. (2005) [55]	- CG: No treatment - IG: Upper limb exercises + diaphragmatic breathing exercises (5 cycles of exercises combined with 1 min rest).	- Upper limb volume - Measurement of extracellular fluids - Tissue resistance to pressure - Subjective upper limb symptoms (pain, heaviness, tension, tingling, burning, perceived size)	- Perimetry - Bioimpedance - Tonometry - MCGill quality of life questionnaire	- Decrease in arm volume at 10 min (% reduction in lymphedema: 5.8%) and maintained for 30 min (*p* = 0.004, 5.3%), 24 h (*p* = 0.04, 4.3%), 1 week (*p* = 0.03, 3.5%) - Volume reduction after one month exercise (*p* = 0.005, 9%) - Decrease in perceived arm size *p* = 0.00 (IG: 4.8 ± 0.2 vs. CG: 5.1 ± 0.4) - Decrease in heaviness *p* = 0.05 (IG: 2.6 ± 0.4 vs. CG: 4.5 ± 0.6)

^1^ CG: control group; ^2^ IG: intervention group; ^3^ VAS: Visual Analog Scale; ^4^ FLI-C: Functional Living Index Cancer; ^5^ VC: vital capacity; ^6^ FEV1: forced expiratory volumen in one second; ^7^ FEV1/FVC: relation between forced expiratory volumen in one second and forced vital capacity; ^8^ MVV: maximal voluntary ventilation; ^9^ TEM: muscle energy technique; ^10^ MIP: maximal inspiratory pressure; ^11^ MEP: maximal expiratory pressure; ^12^ PEP: positive expiratory pressure; ^13^ 6MWT: six-minute walk test; ^14^ FACIT-F: Functional Assessment of Chronic Illness Therapy-Fatigue; ^15^ TMR: Respiratory muscle training; ^16^ MRC: Dyspnea Scale Medical; ^17^ BDI: Baseline Dyspnea Index; ^18^ TDI: Transition Dyspnea Index; ^19^ TUG: Timed Up and Go Test; ^20^ SF36: short-form 36 health survey questionnaire; ^21^ QOL.37: self-administered quality of life questionnaires; ^22^ HFRDIS: Hot Flash Related Daily Interference Scale; ^23^ PCI: Perceived Control over Hot Flashes Index; ^24^ PANAS: Positive and Negative Affect Scale; ^25^ POMS-SF: Profile of Mood States-short form;^26^ PSQI: Pittsburgh Sleep Quality Index; ^27^ STAI: State Trait Anxiety Inventory; ^28^ RSCL: Ro-tterdam Symptom Checklist.

**Table 2:** Quality of Clinical Trials measured with the PEDro Scale.

Author (Year)	Item 1	Item 2	Item 3	Item 4	Item 5	Item 6	Item 7	Item 8	Item 9	Item 10	Item 11	Total
Aybar et al. (2020) [46]	1	1	0	1	0	0	0	1	1	1	1	6/10
Domaszewska et al. (2019) [47]	1	1	0	1	0	0	1	1	1	1	0	6/10
Espinosa-López et al. (2019) [48]	1	0	0	1	0	0	0	1	1	1	1	5/10
Carpenter et al. (2013) [53]	1	0	1	1	1	0	1	1	1	1	0	8/10
Moseley et al. (2005) [55]	1	0	0	1	0	0	0	0	1	1	0	3/10

**Table 3:** Quality assessment of observational studies using the STROBE Statement [51].

Evaluated Section	Item	Ray et al. (2017) [50]	Vilc et al. (2019) [51]	Kulik-Parobczy et al. (2019) [52]
Title and abstract	1	✓		
I: context	2	✓	✓	✓
I: objectives	3	✓	✓	✓
M: study design	4	✓		
M: context	5			✓
M: participants	6	✓	✓	✓
M: outcomes	7	✓		✓
M: data sources/measures	8	✓		✓
M: biases	9	✓		
M: sample size	10			
M: quantitative variables	11	✓		
M: statical methods	12	✓		✓
R: participants	13	✓	✓	✓
R: descriptive data	14	✓	✓	✓
R: outcome variables data	15	✓		✓
R: main results	16	✓		✓
R: other analyses	17			
D: key results	18	✓		✓
D: limitations	19	✓		
D: interpretation	20	✓		✓
D: generability	21	✓		
D: Other information: financing	22			✓

I: Introduction; M: material and methods; R: results; D: discussion.

**Table 4:** Quality of the case studies, as measured by the SCED scale.

Author (year)	Item 1	Item 2	Item 3	Item 4	Item 5	Item 6	Item 7	Item 8	Item 9	Item 10	Item 11	Total
Pedrero-Leal et al. (2019) [49]	1	1	1	1	1	1	0	0	0	1	1	7/10

**Figure A1:**
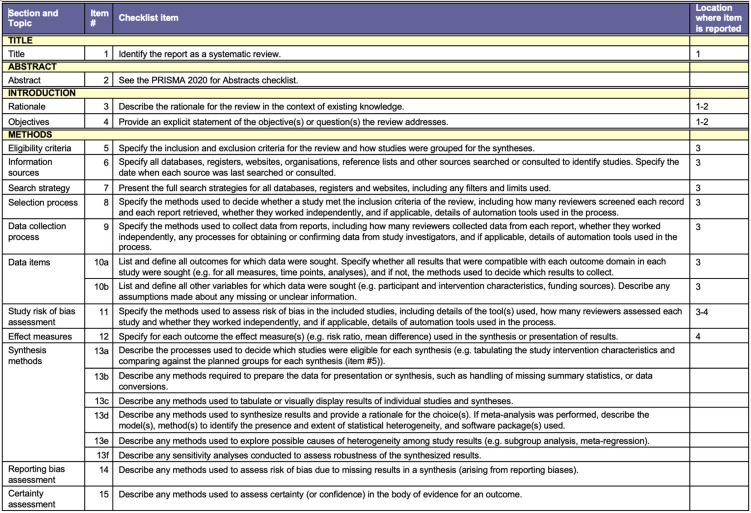
PRISMA Checklist.

**Figure A2:**
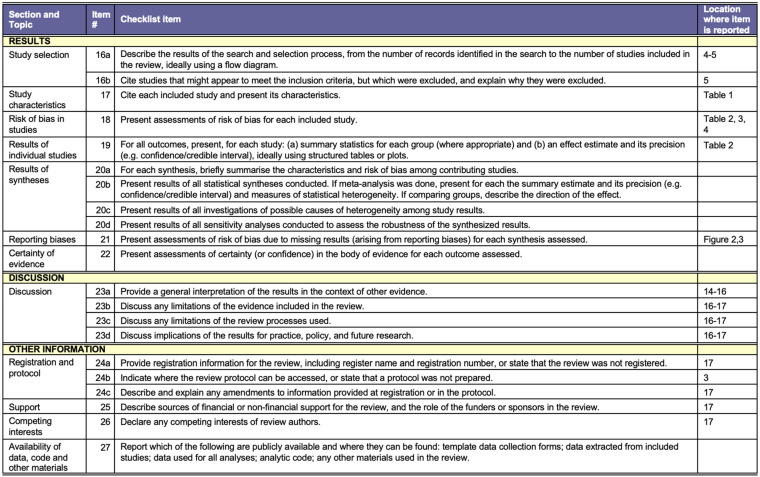
PRISMA Checklist [39].

## Data Availability

Not applicable.
